# Acknowledgment to Reviewers of *Journal of Fungi* in 2021

**DOI:** 10.3390/jof8020106

**Published:** 2022-01-23

**Authors:** Journal of Fungi Editorial Office

**Affiliations:** MDPI AG, St. Alban-Anlage 66, 4052 Basel, Switzerland

Rigorous peer-reviews are the basis of high-quality academic publishing. Thanks to the great efforts of our reviewers, *Journal of Fungi* was able to maintain its standards for the high quality of its published papers. Thanks to the contribution of our reviewers, in 2021, the median time to first decision was 14 days and the median time to publication was 33 days. The editors would like to extend their gratitude and recognition to the following reviewers for their precious time and dedication, regardless of whether the papers they reviewed were finally published:
Aaron NeimanJoy SturtevantAbdelfattah M. SelimJuan Manuel Tovar-PedrazaAbhijit M BalJuan MartínAbid HussainJuan Pablo PardoAbolfazl NarmaniJuan-Miguel Fregeneda-GrandesAbraham Méndez-AlboresJuei-Tang ChengAdalgisa Rodrigues De AndradeJulia PawłowskaAdam OkorskiJulian MuñozAdam PyzikJuliana Alves ParenteAdam ReichJuliana GarciaAdela CmokovaJuliana JunqueiraAdela Martin-VicenteJulie BonhommeAdilia WarrisJulie RibesAdina-Elena SegneanuJuliette GuitardAdrian Gheorghe MartăuJulio A. Massange-SánchezAdriana BlachowiczJulio Alberto ZygadloAdriana M. Rico-RamírezJun OgiharaAgata FabiszewskaJune-Sun YoonAgnes KlarJunichi MaruyamaAgnieszka BęśJunnosuke OtakaAgnieszka MudgeJunyan LiuAgnieszka Piotrowska-CyplikJuraj MedoAgnieszka RichertJürgen HeinischAgostinho CarvalhoJürgen J. HeinischAgustina Ventre-LespiaucqJustyna Karkowska-KuletaAhmad AlshannaqJustyna Lalak-KańczugowskaAhmad MouradJustyna NawrockaAhmed HassanJustyna SulejAhmed LebrihiJyh-fei LiaoAhmed M. Abdel-AzeemKai ZhangAhmed Mohamed Ali KhalilKaitlin ForsbergAhmed NafisKamel A. Abd-ElsalamAhmed S. SultanKamel Abd-ElsalamAida PitarchKamil WojciechowskiAitor Rementeria-RuizKanchan BhardwajAitziber AntoranKandasamy SaravanakumarAize PellonKarl-Heinz RexerAjay Kumar GautamKarl-Henrik LarssonAkira MatsuuraKarolina LabusAkira WatanabeKasun ThambugalaAkos KereszturiKatarina HančevićAlain RivalKatarina VizarovaAlberto Miguel StchigelKatarzyna Otulak-KoziełAlbino Alves DiasKatarzyna PobiegaAlejandro Alvarado-LassmanKatarzyna SkrzypczakAlejandro De Las Peñas NavaKatarzyna WińskaAlejandro Hernández-LeónKateryna DavydenkoAlejandro Rodriguez-SanchezKatia BoggianAles EichmeierKatie M. OhnoAlessandro BuscaKausik DattaAlexander BetekhtinKavita SharmaAlexander F.A.D. SchauwvliegheKazuhisa SekimizuAlexander IgnatovKelly IshidaAlexander LichiusKen OdaAlexander NaumkinKenji KitamuraAlexander OrdynetsKennio PaimAlexandra A. PopovaKeunje YooAlexandra SolomouKeyvan PakshirAlexandre LamasKi Choon ChoiAlexandre MaréchalKiessoun KonatéAlexandre Melo BailãoKinan AlhallakAlexandro BonifazKinan Drak AlsibaiAlfons JM DebetsKirchmair MartinAlfonso Fernández-ÁlvarezKirk M. DrueyAlfred O. AnkrahKirsten N. NielsenAlfredo GueraKishneth PalanivelooAli AbdallahKlarić Maja ŠegvićAli NawazKoichiro MoriAli NomanKonstantin ChekanovAli Rezaei-MatehkolaeiKonstantin V. MoiseenkoAlia SagatovaKris VernelenAlicia Gomez-LopezKristiina HildenAlicia Moreno-SabaterKsenija DurgoAlida SorrentinoKwaku KyeremehAlina AlexandrovaKwang-Soo ShinAlina Kunicka-StyczyńskaKwang-Woo JungAlla SplichalovaL. AravindAllan Patrick G. MacabeoLabuda RomanAllen BrownLahcen OuahmaneÁlvaro Rodríguez GonzálezLakkakula SatishAmalia AnastasopoulouLarissa FernandesAmanda CastoLarissa KrasnopolskayaAmariliz RiveraLászló Kozma-BognárAmelia Portillo LópezLászló MajorosAmer Hayat KhanLaura BurrackAmir ArastehfarLaura GarzoliAmira YacoubLaura GiamperiAmnart PoapolathepLaura MugnaiAmolkumar U. SolankeLaura MurciaAmr ElKelishLaura Naemi WaltiAna C. SampaioLaurent DufosséAna Camila Oliveira SouzaLaurie B. ConnellAna Cristina EstevesLavinia RutaAna Espinel-IngroffLawrence W. ZettlerAna Fernandez-CruzLay Sun MaAna Lúcia GonçalvesLecturer Carmen Rodica PopAna Maria PovedaLei CaiAna Oliveira SouzaLeire Molinero RuizAna PastranaLeire Molinero-RuizAna TomasLena StudtAnand Anand RamasubramanianLeonard AtanaseAnani K. AfanouLeonora Mendoza EspinolaAnastasia AntoniadouLeticia Bernal-MartínezAnastasia SaadeLetizia AngiolellaAnca Livia Butiuc-KeulLiao Hung-JuAnders JohansenLiat Ashkenazi-HoffnungAndré FleißnerLidia BłaszczykAndré Luis Souza Dos SantosLidia Hanna MarkiewiczAndré NicolaLiliana Aranha CaetanoAndrea ArdizzoniLiliana Oliveira RochaAndrea BecchimanziLiliana SantosAndrea CortegianiLilyAnn Novak-FrazerAndrea KunovaLinda HansonAndrea RubiniLinda S HuangAndreas GrollLindsay K NewboldAndreas H. GrollLinghong MengAndres MäeLiping SunAndrew D. CartmillLiqiong GuoAndrew H. LoudonLisa LombardiAndrew M. BormanLise Nistrup JørgensenAndrew MillerLi-Wei ZhouAndrew R. GenneryLóránt HatvaniAndrey R. SuprunLouise A. WalkerAnelize BauermeisterLouise Egerton-WarburtonAneta PtaszynskaLoukas KanetisÁngel BenítezLubos CipakAngela CapeceLuca RuiuAngeliki AndrianakiLucia ČernákováAngelo GismondiLucïa RamírezAngelo Maria GiuffrèLucia SilvestriniAngie GelliLudger StändkerAnis LimamiLudmila Matos BaltazarAnita BiesiadaLudwika Tomaszewska-HetmanAnita GomesLuigi De BellisAnita HaegiLuigi Di BitontoAnita KlausLuigi PrincipeAnja ForcheLuigi SantacroceAnke BurmesterLuís AbrunhosaAnket SharmaLuis Cubilla-RiosAnn PackeuLuis PocasangreAnna GiammancoLuisa MirandaAnna KotŁukasz StępieńAnna MuszewskaLuke CurtisAnna PoliLuke LatimerAnna Szafranek-NakoniecznaLysett WagnerAnna Zimoch-KorzyckaM. José García IglesiasAnnamária PallagM Lourdes AbarcaAnne CosteMaciej BoguńAnne-Cécile NormandMaciej JaneczekAnne-Marie Crutz-Le CoqMacit IlkitAnnette JensenMadhu KamleAnoop AlexMagalie DemarAnsgar PoetschMagdalena CalAntoine AdenisMagdalena SzczechAntonietta MelloMahboob AlamAntonio CassoneMahdi AbastabarAntónio InêsMahmoud S.M. MohamedAntonio Maria FeaMaiken ArendrupAntonio Peña DíazMaja KaramanAntonio PisabarroMałgorzata GrabarczykAntonio PortugalMałgorzata GumiennaAntonio RossiMałgorzata SztankeAntonios N. PapadopoulosMallique QaderAntonius R B OlaMamatha BallalAnup K GhoshMan Shun FuAnuradha ChowdharyManas Kumar BagAnurag SinghManish DubeyAnurag SunpapaoManu KumarAnutthaman ParthasarathyManuela OliveiraAparecido SouzaMara ParaskeviArash ZibaeeMara QuagliaArianna TavantiMarc StadlerArlete Mendes FaiaMarcel PârvuArmida Sánchez-EscalanteMarcela AyalaArmin DjameiMarcello TagliaviaArnab GhoshMarcelo Marucci Pereira TangerinaArnaldo Lopes ColomboMárcia MelhemArnaldo VideiraMarcia SaraivaÁrpád CserneticsMarcin BryłaArthur KruckebergMarcin PietrasArun DuttaMarcos MoraesAsaf SalamovMarek BartaAshish A PrabhuMarek KieliszekAshley GarrillMargarida DuarteAshraf S. IbrahimMargarida PalmaAshwini Khanderao JadhavMargarita Canales-MartinezAsma OsivandMargherita ScapaticciAssaf Arie BargMaria A. AnderssonAsta JudzentieneMaria A. LongoAstrid K. LeckMaria AntinoriAthanasios TragiannidisMaria AragonaAthanasios ZervasMaría BuitragoAtmika PaudelMaria Da Gloria Teixeira De SousaAtsushi IwataMaría Del Mar HernándezAuke De JongMaría Del Rayo SánchezAurora StorlazziMaría E. Morán-DiezAvishek MitraMaria ExindariAyelen TayaguiMaria Filomena CaeiroAzam FattahiMaría Florencia ViannaBachir BenarbaMaría Francisca Colom ValienteBaojun WuMaria G. SamsonovaBaoye HeMaría Guadalupe Frías-De-LeónBarbara BlackwellMaria Isabella PrigigalloBarbara FrąszczakMaría Luján CuestasBarbara WiewióraMaria N. GamaletsouBarry SavilleMaria Navarro-MendozaBartłomiej ZieniukMaria NoniBeata Guzow-KrzemińskaMaria Rapala-KozikBeatrice BelfioriMaria Sameiro T. GonçalvesBeatriz Sevilla-MoránMariaelvina SalaBelaghihalli N. GnaneshMarianna CaterinoBenedetto T. LinaldedduMariano Sánchez CrespoBenjamin HorwitzMaribel PortillaBernadette TelekyMarie Desnos-OllivierBeth A. GarvyMarie DufresneBeth FuchsMarie-Noelle RossoBilal ÖkmenMarika PellegriniBingda SunMarina KostićBirgit WillingerMarina MachadoBi-Shuang ChenMarina Marcet-HoibenBogdan Gabriel ȘlencuMarina SokovicBojan ŽunarMarina V. PadkinaBoredi Silas ChidiMarine ValletBożena Pawlikowska-PawlęgaMario CrucianiBożena StodolakMario MasielloBrenda WingfieldMario VendittiBridget BarkerMariolina BrunoBruce DienMarion L. WoodsBryn ShortMarion MillotBurragoni Sravanthi GoudMaris LaubertsByungsuk KwonMaristella MastoreC. Patrick LuskMariusz DylagCaihong DongMariusz GagośCal Antonieta DeMarjatta RaudaskoskiCameron K. TebbiMark FraserCameron R. CurrieMark KrockenbergerCampbell GourlayMark SeawardCandice CoombesMark WillcoxCarla Huarte-BonnetMarlena Baranowska-WasilewskaCarla PalmaMarta De ZottiCarla RagoneziMarta HerreraCarlos Perez ArquesMarta J. FiołkaCarlton Ralph C. DanielMarta LaranjoCarmela Maria MontoneMarta OlechCarmelo BiondoMarta TuronCarmen Gómez-Lama CabanásMartha Marie VaughanCarmen MichánMartin KelloCarmine GuarinoMartin SchmidtCarol KauffmanMartin ValachovicCarol MeteyerMartyna MroczyńskaCarolina CoelhoMarwan M. AzarCarolina G. PoitevinMarzia VergineCaroline MarcosMarzieh AlizadehCaroline PoyntnerMasahiro AbeCaroline StrubMasanori SaitoCassamo Ussemane MussagyMasao HigoCassian SitaruMasoomeh Ghobad-NejhadCathrien A. EgginkMasoume AmirkhaniCathy CrippsMatan ShelomiCécile GueidanMateusz WilkCécile LorrainMathieu NacherCécile NeuvégliseMathieu RouardCesar RonceroMathu C. MalarCezary TkaczukMatthew LebarChadi A. HageMatthew P. NelsenChangLin ZhaoMatthias HahnChanglong ShuMatthias KretschmerChangqi LiuMaud Gits-MuselliChantal FradinMauricio Soto-SuárezChao-Xi LuoMax Carlos Ramírez-SotoCheng JinMayanka AwasthiChengmiao YinMayura VeeranaCheol-Won YunMazin BarryCheuk Lun LiuMd. Rabiul IslamChiara DaccòMedhat MahmoudChien-Ming ChaoMegan RombergChien-Sen LiaoMehdi Nasr IsfahaniChih-Chia SuMehdi SoltaniChing-Fu LeeMeisam TabatabaeiChin-Soon PhanMekala VenkatachalamChin-Tung WuMélanie IkehChris KosmidisMelinda FogarasiChristian AndrighettoMeritxell Riquelme PérezChristian PaetzMerrick EkinsChristiane BaschienMethee ChayakulkeereeChristina C. ChangMichael A. PfallerChristina CuomoMichael ArabatzisChristine BonnalMichael E. ZegansChristine ImbertMichael G. WellerChristoph SchüllerMichael Hilary OtimChristophe CamusMichael J. BradshawChristophe HennequinMichael McMurrayChristophe PadoinMichael OverduinChristopher NileMichael PalmerChristos D. GeorgiouMichael PerlinChung-Yao HsuMichael ReidyClaire PriceMichaela LacknerClara BaldinMichail LionakisClara Martínez-AriasMichal ChomaClarisse BrígidoMichał PiegzaClark L. OvreboMichel AlmaguerClaude PlassardMichel MonodClaudia ColeineMiguel A. PeñalvaClaudia R. Bonini-DomingosMiguel MatillaClaudia RiccioniMiguel Ponce-VargasClaudia SimmMihai NeteaClaudio CaprariMiklós NagyClaudio FarinaMikušová PetraClaudy Oliveira Dos SantosMila GrahovacClement K.M. TsuiMila SantosColin J. InghamMilena KordalewskaCong JiangMing-Cheng WuCongqiang ZhangMing-Horng TsaiConstantin UrbanMing-Tse KuoCormac MurphyMingxia ZhaoCotica Erika Seki KioshimaMingyu HeCraig FauldsMingyuan WangCristina Burrola-AguilarMinh Vien LeD. AntonetsMin-Ji KimDaeui ParkMinoru YoshidaDafne BongiornoMirela Ivančić ŠantekDamijan KnezMirela OsrečakDaniel AgustinhoMiriam Alisa KnollDaniel DobslawMiriam HaidukowskiDaniel EladMiriam Van Den NestDaniel HenkMirko CucinaDaniel P. PotaczekMiroslav CaboňDaniel Palmero-LlamasMiroslava KačániováDaniel R. LauciricaMiroslava PožgajováDaniel SchindlerMirza HasanuzzamanDaniel Zamith-MirandaMitsuru TodaDaniela ClericiMohamed A. El-EsawiDaniela De VitaMohamed A. HassanDaniela IsolaMohamed Al-FatimiDaniela JakšićMohamed El HattabDaniela MaroneMohamed El-ShetehyDaniele Roberto GiacobbeMohamed F. AbdallahDanilo ThomasMohamed Fathi AbdallahDanny HaelewatersMohamed ShaabanDanon Clemes CardosoMohammad Javad NajafzadehDanuta NowickaMohammad SayariDaren W. BrownMohammadreza SalehiDaria AugustyniakMohammed A. A. El-KholyDaria ChlebekMohammed Rafiq H. SiddiquiDarin WiesnerMohan KaruppayilDariusz LatowskiMohd Hafiz ArzmiDavid AnguloMohit BibraDavid CahillMohsen ElsharkawyDavid CánovasMojgan RabieyDavid E. LevinMonica AgnolucciDavid García De LeónMónica GalochaDavid Macaya-SanzMónica GandíaDavid RondeauMónica Lamas MaceirasDavid ŠilhaMónika BálintDavid W. DenningMonika Kadela-TomanekDawid MikulskiMonika SommerhalterDawid StefaniukMorris MaduroDea Garcia-HermosoMostafa RatebDeanna H. OlsonMotoi TakenakaDebasish Kumar DeyM.P. SharmaDejan StojkovićM.Teresa Martin-GomezDelgado JosueMuhammad AzamDenes DlauchyMuhammad KashifDenis KorneevMukesh MeenaDenis RusjanMuneyoshi Kanaider Nest Magriet vanMurali MaruthamuthuDe-Wei LiMurugesan ChandrasekaranDhamodharan VenugopalMurugesan VanathiDhanushka N. WanasingheMustafa SevindikDiana MachadoMuthu ThiruvengadamDiego H. CáceresMuthugounder Subaramanian ShivakumarDiego LunaNadezhda V. PsurtsevaDiego QuirogaNaeem KhanDieter WolfNalin WijayawardeneDiletta RosatiNalu T. A. PeresDimitri PoddigheNancy Weiland-BräuerDimuthu S. ManamgodaNaotake KonnoDinesh RokayaNaoyoshi KumakuraDivya GopinathNarayan Chandra PaulDmitry KarpovNatalia A. PeresDolunay GülmezNatalia Mendoza-PalomarDomenico DavolosNatalia Nowacka-JechalkeDomenico GiosaNatalia V. BeloborodovaDomenico PrisaNatasa BozicDonald NatvigNataša ŠtajnerDonald O. NatvigNathan C. BahrDonatella TeseiNeal BhatiaDonato MagistàNeil GowDong-Kyu KimNelson Barros ColautoDora E. Corzo-LeónNeringa RasiukeviciuteDorina PodarNessim KichikDouglas F. LakeNevena GrdovićDuc Cuong BuiNicola De SimoneDulce Maria Palmerin- CarreñoNicola LandiDunja ŠamecNicola WannickeEckart HanekeNicolás PedriniEddie G. DominguezNicolau SbarainiEdeltrudes De Oliveira LimaNicoleta Nicole RaduEdgar CambazaNikolaos KatzilakisEdson Rodrigues-FilhoNikos TzortzakisEduardo A. EspesoNishad Thamban ChandrikaEdward SionovNitipong PermpalungEelco F. J. MeijerNor Hasmaliana Abdul ManasEfstratios Stratos NikolaivitsNorlia MahrorEkaterina BaranovaNorman Van RhijnEkaterina SkolotnevaNuria Álvarez-SánchezEkaterina YurchenkoOadi MatnyEkram HossainOfer GoverElah PickOgueri NwaiwuElaine Cristina FranciscoOier EtxebesteElda RighiOksana LastochkinaEléna CharpentierOladapo BakareElena PieckovaOlaf SchmidtEliana Barreto-BergterOlga A. GlazunovaEliane Pereira CipolattiOlga Rivero-MenendezElias AlisaacOlga V EfremenkovaElisa González-DomínguezOlga ZimmermannováElisabeth JacobsenOlimpia LaiElisabetta GrilloOliver KurzaiElizabeth AitkenOlivier GoncalvesElizabeth CzislowskiOlivier Van WuytswinkelElizabeth K. BrauerOmar HarbElpis MantadakisOndrej CehlárEl-Saadony MohamedOndrej KoukolElsherbiny ElsherbinyÓscar González-LópezElżbieta PatkowskaOtto MiettinenEmanuel VamanuP. Murali KrishnaEmilie BruezPanagiotis A. EliopoulosEmilie FréallePankaj PandeyEmilie WidemannPao Theen SeeEmma GachomoPaola AngeliniEmmanuel BertrandPaola MuggeoEmmanuel WickerParés-Matos ElsieEncarnación Pérez ArtésParissa TaheriEnrica MatteucciPasquale RussoEnrique J. CalderónPat WolseleyEnrique Quesada MoragaPatrícia AlbuquerqueE.P. BarantsevichPatricia EscandónErez Bar-HaimPatricia Gomes CardosoEric A. GriffinPatrícia H. BritoEric JohnsonPatricia L. Lakin-ThomasEric McKenziePatricia PolettoEric RecordPatricia PriceErica Elisa FerrandiPatricia Tamez-GuerraErica LuminiPatricio RamosErick Martínez HerreraPatrick SchwarzEricsson Coy-BarreraPatrizia DanesiErika KothePaul HooykaasErika ShorPaula GonçalvesErika SoldiPaula JouhtenErika SutantoPavel MatušinskýErin McClellandPavel SemenyukErin RehrigPaweł GutErmelinda SilvaPawel KafarskiEusebio NavarroPedro A. Jiménez GómezEustachio TarascoPedro GonçalvesEva ČonkováPedro PaisEva María Gómez OrtePedro TalhinhasEvelina TutucciPei-Cheng HuangEvelyne SelberherrPeter GeiduschekEvgenia PapakonstantinouPeter J JudgeEwa Beata GórskaPeter OelkersEwa Dorota KrólPeter PolčicEwa RopelewskaPetros IoannouEwelina PatyraPhilippe SilarF. Conceição SilvaPiazza Simone DiFabiana CaniniPierre BeckerFabienne MalagnacPierre LapaquetteFabrício Ávila RodriguesPi-Han WangFabrizio BeltramettiPiia KarisolaFang LiuPin-Der DuhFang YouPiotr B. HeczkoFarley William Souza SilvaPiotr BilańskiFatima El KhalloufiPiotr HapetaFederica SpinaPiotr JuszczykFederico VitaPooja SinghFelipe H. Santiago-TiradoPopchai NgamskulrungrojFelix BongominPrachand ShresthaFerenc BagiPradeep KumarFerenc OroszPranshu SahgalFernanda BadottiPrasun KumarFernanda MonedeiroPratyush RoutrayFernanda MorgadoPrem Lal KashyapFernanda WirthPrem Pratap SinghFideline Laure Tchuenbou-MagaiaPruthvi Balachandra KalyandurgFilippe Elias De Freitas SoaresQazi Mohd Rizwanul HaqFlorence PersatQian ChenFlorian AltegoerR. K. Murali BaskaranFlorian HennickeRachid LahlaliFlorin Daniel LipsaRadivoje JevtićFortunato Palma EspositoRadu Claudiu FierascuFrancesca BeriniRafael José Vilela De OliveiraFrancesca DegolaRafał OgórekFrancesca GarganeseRafal SawickiFrancesca ManciantiRaffaele CarrieriFrancesco BarchiesiRaffaella Maria BalestriniFrancesco CalzaranoRahul Mahadev ShelakeFrancesco Di GennaroRajagopal SubramaniamFrancisco ChavezRajneesh JaswalFrancisco EpeldeRam PrasadFrancisco J. GeaRaman JegadeeshFrancisco J MedranoRamesh VetukuriFrancisco SalinasRamón Alberto Batista-GarcíaFrancisco SolanoRandy DinkinsFranck PanabieresRaphaela De Castro GeorgFrank SchurenRaquel AtxaerandioFranscesco BarchiesiRaquel Fernandez-GarciaFrederic LamothRaquel MayordomoGábor KardosRaquel Pino-BodasGábor NagyRebeca Alonso-MongeGabriele BiancoRebecca CreamerGaëtan L.A. MislinRebecca DrummondGanesan A.Rebecca SweanyGanesh SarataleReeta Rani SinghaniaGang LiRegina Helena PiresGarcia-Rubio RocioRenata TeparicGaurav BairwaRenata TisiGautam AnandRenata VadkertiovaGeelsu HwangRenato KovacsGennaro AgrimiRency VargheseGeorge DeepeRenuka N. AttanayakeGeorge DimopoulosRicardo Sánchez-CruzGeorgia OuzounidouRiccardo Zenezini ChiozziGeorgia VrioniRichard Alan HumberGeorgios ChamilosRichard CalderoneGerald BillsRichard CannonGerardo Vázquez-MarrufoRichard J. BennettGiacomo AndreaniRichard MalikGiacomo CasaliniRichard SeigelGiampaolo SimoniniRigoberto Hernández-CastroGiancarlo PerroneRiina RichardsonGiannini Maria MendesRiko HatakeyamaGill DiamondRimvydas VasaitisGilles NevezRiyazuddin RiyazuddinGina HongRobbert BentvelsenGina WallRobert ArkowitzGiorgia PertileRobert KrauseGiorgio MaresiRobert RileyGiovanna SimonettiRobert RusinekGiovanna SiracusaRobert Warren BehleGiovanni BubiciRobert WheelerGloria M. GonzálezRobert ZarnowskiGonzalo G. BarberáRoberta GaluppiGrant T. KirkerRoberto BaroneGregorio VonoRoberto Fernandez-LafuenteGretty VillenaRoberto Montesinos-MatíasGrzegorz JanuszRobin SebastianGrzegorz LemańczykRobin WilganGuillaume BilodeauRocio Garcia-RubioGuillaume J. BilodeauRocio MedinaGuillermo Garcia-EffronRoderick HayGuillermo QuindosRodolfo ParadaGunther DoehlemannRodrigo Rollin-PinheiroGustavo A. Niño-VegaRohit BazazGyöngyvér MaraRohit SrivastavaHamed BostanRok TkavcHan WöstenRoman CrazzolaraHana SevcikovaRoman NowickiHans-Joachim SchüllerRonald E. GarnerHans-Josef SchroersRonen Ben-AmiHao-Xun ChangRong-San JiangHari SudiniRonit SionovHarold J G MeijerRonnie WillaertHassan MahmoudiRonny MartinHayato MasuyaRony SwennenHeather ContiRory WelshHeather RumbleRosa HermosaHector Mora-MontesRosa SacedónHee-Soo ParkRosanna TofaloHelen TreichelRosario Medel OrtizHelena BujdákováRose MartinHelena Gil AzinheiraRossana De Aguiar CordeiroHema Prasad NarraRoxana VidicanHemant K. MishraRoy A. KhalafHeng GuiRoy KennedyHenk-jan SchoonbeekRuben A. AbrantesHenri MaraiteRuben Manuel Luciano Colunga BiancatelliHernández-Domínguez Eric EdmundoRuilong ShengHervé Van Der HeydenRungroch SungthongHideyuki KawauchiRuvishika JayawardenaHillary RighiniRyan BracewellHiran AriyawansaRyo OhtomoHiroshi KitagakiS. Karthick Raja NamasivayamHirotomo KatoSaad El-Din HassanHisashi NishiwakiSabine Dagmar ZimmermanHoracio BachSabine StrahlHortencia Silva-JímenezSadanand PandeyHossein AhangariSadri ZnaidiHubert SytykiewiczSajeewa MaharachchikumburaHugo P. AdamoSalleh AnnasHung-Yin LinSalman HussainHyang Burm LeeSalomé LeibundGut-LandmannHyeon-Su RoSalvador MireteHyeri SeokSamantha C. KarunarathnaHyunkyu SangSamantha KarunarathnaIan ClearySambasivam PeriyannanIchiro KameiSánchez-Rangel DianaIdzi SiatkowskiSándor GondaIgor A. KazartsevSándor KocsubéIgor MorgunovSandra MartinsIgor N. PavlovSandra Moraes Gimenes BoscoIker Falces-RomeroSandra SavocchiaIleana Andreea RaţiuSandrina HelenoIleana C. FarcasanuSanford David VanImre HolbSanja TibellInês CorreiaSanjay G. RevankarInês DinizSanta O. CacciolaInhyung LeeSanta Olga CacciolaInmaculada Garrido-JuradoSantiago Sánchez-RamírezInmaculada VacaSantos José C.S. dosIoanna PyrriSantosh GhoshIoannis KourkoutasSaowaluck TibprommaIoannis KyriakidisSara GagoIoannis-Dimosthenis AdamakisSara Mayo-PrietoIoly Kotta-LoizouSarah DellièreIosif MarincuSarah KykerIqbal NousheenSarah Santos GonçalvesIrene StefaniniSarath GutiérrezIrene ValasiSaul FrenkielIrina LyanguzovaSaurav Kumar JhaIrlon Maciel FerreiraScott FillerIrmgard Krisai-GreilhuberSean D. MooreIrshad SharafutdinovSebastian GnatIsabel A. C. RibeiroSebastian PiłsykIsabel Antonieta Iturrieta-GonzálezSébastien ImbertIsabel MartinsSengodan KarthiIsabella GrishkanSeraphim PapanikolaouIsidro Gonzalez-ColladoSerge AlfandariIsrael Enrique Padilla-GuerreroSergey KarpovIvan MihálSergi Garcia-BarredaIvan SacheSergi MaicasIvan ŠirićSergio Sánchez PeñaIvana MarekovićSeri RobinsonIvana Tlak GajgerSeverine MatthijsIvan-Kresimir SvetecSezai ErcisliIwona SembratowiczShagufta PerveenIzabela ZakrockaShakeel Ahmad KhanJ. Calvin KouokamShamsul BhuiyanJ. Carlos IgualShang Yi LinJacek C SzepietowskiShannon EsherJacek KominekShawn LockhartJacek KozdrójShigeaki SaithoJacopo CalevoShigeki SetoJadwiga WyszkowskaShigemi SeoJaime CarrascoShigeru MorimuraJair PutzkeShigeyuki TanakaJairo Castaño-ZapataShivankar AgrawalJairo LizarazoShivaprakash RudramurthyJakub SuchodolskiShohei MitaniJalal Jalali SendiShunping DingJames BreretonShuwen DengJames HurleySilvia Salas-PinoJames KonopkaSimon SchiwekJames L. KlotzSimona DzurendovaJames RiddellSimone CantamessaJames T. MorrisSimone CesaroJan KiebistSiraprapa BrooksJan StyczyńskiSlavica JanevskaJan VondrákSlavomír AdamčíkJan ZollSławomir MilewskiJana ČopíkováSlimane KhayiJane K. DeverSofia AgriopoulouJane UsherSofia Costa-de-OliveiraJanis LiepinsSofia MavrikouJarmila CelakovskaSomanon BhattacharyaJaroslav PejchalSoo LeeJarosław GrządzielSoon Gyu HongJarosław L. PrzybyłSophie BrunJasmina GlamočlijaSophie LorberJason BrownSophie TrouvelotJason StewartSophon BoonlueJassy DrakulicSotiris AmillisJavid Ahmad ParraySowmyalakshmi SubramanianJavier Lopez-CeperoSpinello AntinoriJavier MoragaSpiridon MantzoukasJavier PérezSpiros ParamythiotisJay K. KollsSrinivasan RamalingamJean MenottiSrinivasan SathiyarajJean Pierre GangneuxStanislav TrdanJean SavoieStanislava VoběrkováJean-Bosco JoudaStefan D. MomčilovićJean-Michel SavoieStefan ShilevJeannina SmithStefan SimmJeffrey BaraStefania ContiJeffrey ColemanStefania MangJeffrey M. LorchStefania ZanettiJelena LozoStefano MartellosJeniel NettStéphane BretagneJens FrisvadStephen FreeJentaie ShieaSteve LeavittJen-Tsung ChenSteven D. HarrisJeonghwan JangSteven HarrisJersson PlacidoSteven T. DenhamJessica C. BrownSudeep TiwariJessica M LohmarSujiraphong PharkjaksuJessie FernandezSujit JagtapJessie K UehlingSujit ShahJesús AguirreSukhvinder SinghJesus Martín-GilSumio IshijimaJianping XuSun-bean KimJin ZhangSusan FrankelJinrong XuSusan P. McCormickJiří ČerveňSusumu KawakamiJiří NermutSuzana OtaševićJitendra Kumar TripathiSuzy MoodyJiunn-Liang ChenSven HerzogJoana RodriguesSvetlana DinićJoana RoloSvetlana KamzolovaJoanna DłużniewskaSylwia StączekJoanna Gromadzka-OstrowskaSzabolcs RudnóyJoanna KaminskaTaiki FutagamiJoanna KruszewskaTakashi KanbayashiJoanna StefanskaTakashi MatsumuraJoanna Żur-PińskaTakayoshi AwakawaJoao Ricardo AlmeidaTakehiko KanazawaJoão TrovãoTania RaymundoJoaquin ArinoTariq MukhtarJochem B. BuilTatiana Robledo-MahónJoel M. FriedmanTatiana S. FrolovaJoerg SteinmannTatyana PasechnikJohannes BallauffTeixeira Miguel CachoJohannes FreitagTewodros MulugetaJohannes WöstemeyerThatsanee LuangharnJohan-Owen De CraeneThierry RollingJohn A JohnsonThorsten KraskaJohn E. BennettTimeleon VizantiadisJohn F. KernienTsutomu ArieJohn Paul Délano-FrierTung-Yi LinJolanta Behnke-BorowczykUlrich SinschJolanta MierzejewskaUrsula FleigJon P. WoodsVahid FarzanehJon Salmanton-GarcíaValentina Arsic ArsenijevicJong Soo ChoiVan Khanh NguyenJoon-hoon KimVincent BrunoJordy CoolenViorica Railean-PlugaruJorge BarriusoVishnu D. RajputJorge L. Folch-MallolWagner Luiz BatistaJorge Martínez QuesadaWalter P. PflieglerJorge PovedaWerner HeinzJorunn BörveWolfram MittelmeierJosé A. CañasWonyong KimJosé Arturo Molina-MoraXiang SunJosé IbeasXingzhong LiuJosé Manuel CifriánYang BiJosé Pedro Guirao-AbadYang Jae KangJose RamosYeon Soo HanJose ThekkiniathYiming LiJoseph F. AmmiratiYoshihiro KobaeJoseph StraussYoshinori ArisakaJosephine WeeYuichi MineJoshua J. ObarYukihiro HamadaJoshua NosanchukZhu-Liang YangJosué J. Da SilvaZoltán BaráthJovana PetrovićZoltán BratekJoveria FarooqiZuzana Hruska


